# Characterization of the organelle genomes of mycoheterotrophic *Thismia angustimitra* and evidence suggesting horizontal gene transfer

**DOI:** 10.1186/s12864-026-13542-9

**Published:** 2026-11-05

**Authors:** Gitte Petersen, Henrik Ærenlund Pedersen

**Affiliations:** 1 Department of Ecology, Environment and Plant Sciences, Stockholm University, Stockholm, 106 91 Sweden; 2 Natural History Museum of Denmark, University of Copenhagen, Copenhagen, Denmark

**Keywords:** Horizontal gene transfer, Mitogenome, MTPT, Mycoheterotrophy, Plastome, Recombination, RNA editing, *Thismia*

## Abstract

**Background:**

Our knowledge about mitochondrial genome evolution in plants is lacking far behind our knowledge of plastome evolution. In heterotrophic plants, it is well-known that plastome degradation and reduced or lost photosynthesis go hand in hand, and there is little if any difference between plastome evolution in mycoheterotrophic and haustorial parasites. The mitochondrial genome appears less affected by a heterotrophic lifestyle, although exceptions occur. In contrast to plastome evolution, mitochondrial evolution may differ between mycoheterotrophic and haustorial parasites, in particular with regards to the occurrence of horizontal gene transfer. However, data from mycoheterotrophs are scarce, and complete mitogenomes are only known from three of ten clades.

**Results:**

Here we present data from the complete plastome and mitogenome from *Thismia angustimitra*, representing a fourth clade of mycoheterotrophs. The 18,215 bp plastome is much reduced and includes only few genes. The mitogenome has a multicircular structure consisting of 22 chromosomes with a total length of 422,400 bp. The gene content and the pattern of RNA editing are normal. The mitogenome includes only few repeats and recombinational activity is limited. A search for foreign DNA did not reveal any fungal sequences, the content of plastid DNA is low, but a large proportion of intergenic DNA shows similarity to distantly related angiosperms. Just a few plastid inserts appear to have originated through intracellular transfer, whereas others most likely stem from horizontal gene transfer. Synteny patterns of the intergenic sequences suggest that horizontal transfer may happen in either direction.

**Conclusion:**

The highly reduced plastome of *T. angustimitra* fits completely the general picture of plastome degradation in heterotrophic plants. Whereas the mitogenome in many aspects appears unaffected by the lifestyle, the recombinational activity is unusually low. Surprisingly, we find evidence suggesting horizontal gene transfer, indicating that this phenomenon may be more common among mycoheterotrophic plants than previously thought.

**Supplementary information:**

The online version contains supplementary material available at https://doi.org/10.1186/s12864-026-13542-9.

## Background

**Fig. 1:**
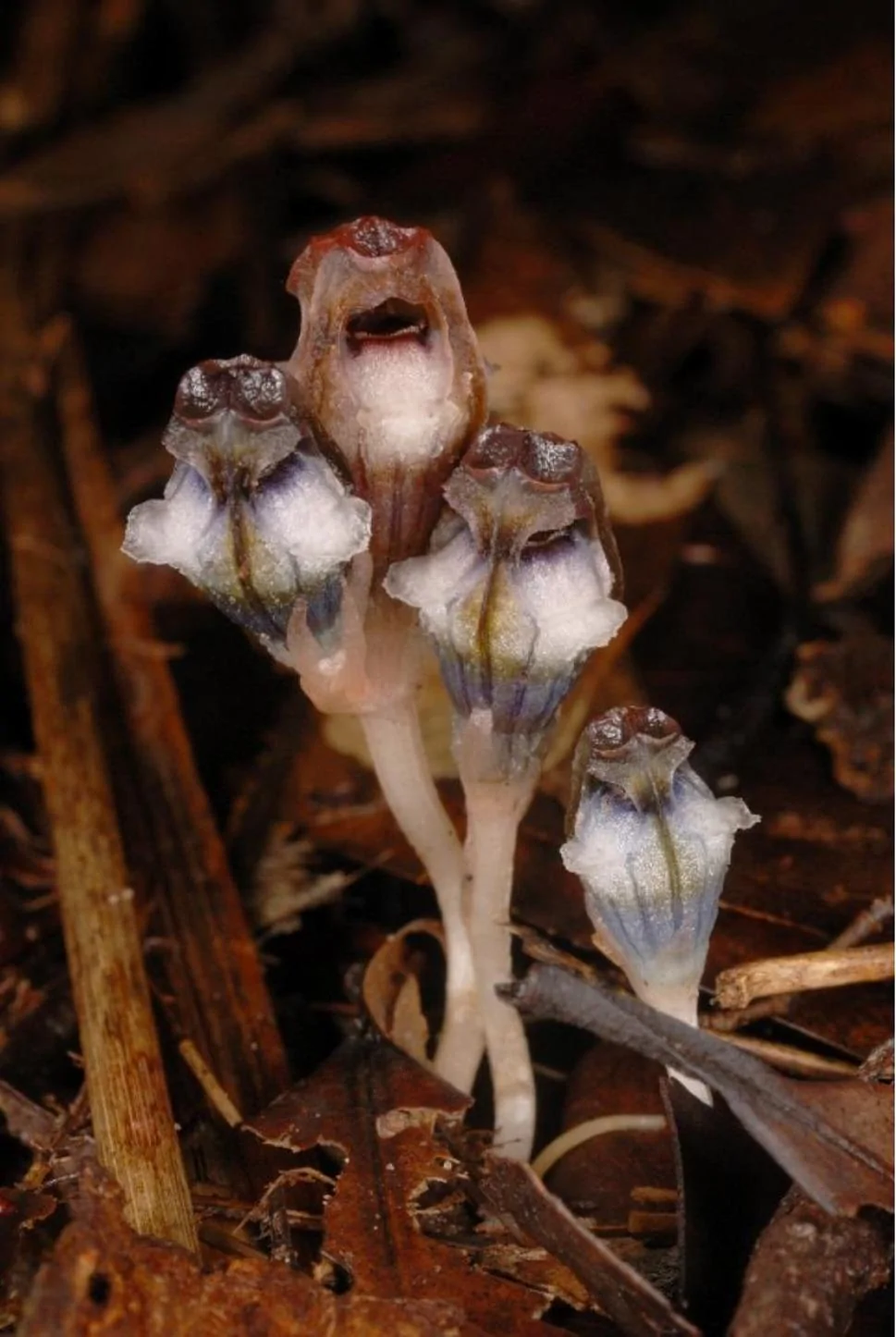
*Thismia angustimitra* photographed at the type locality in Buengkan Province of north-eastern Thailand, where also the DNA sample was collected. Photograph H. Æ. Pedersen

The genus *Thismia* (Thismiaceae, Dioscoreales) comprise more than 100 species, and new species are still being described [1]. They are minute, mycoheterotrophic and achlorophyllous plants with a peculiar floral morphology and are known as “fairy lanterns” (Fig. 1).

It is well known that the plastome of mycoheterotrophic and other parasitic plants having lost photosynthesis is highly degraded [2, 3]. Plastomes of *Thismia* and other related species of Thismiaceae are no exception. They are much reduced in size, typically some 15–20 kb, some have lost the inverted repeat (IR), and the gene content is limited to housekeeping genes [4–7]. In *Thismia* only ribosomal protein coding genes, rRNA genes, tRNA genes, and *accD* are left, with the total number of presumably functional genes varying among species from seven to 15 [5].

The mitochondrial genome (the mitogenome) is expected to be far less affected by a mycoheterotrophic and achlorophyllous lifestyle [8]. The genes primarily being involved in cellular respiration are still needed, although loss has occurred in parasitic but photosynthetic mistletoes (*Viscum* and other Viscaceae) [9–11]. However, complete mitogenome data from mycoheterotrophic plants are still scarce. Mycoheterotrophy has evolved in 10 flowering plant families, but complete mitogenomes have only been assembled from *Monotropa* in Ericaceae [12], *Epirixanthes* in Polygalaceae [13], and three genera (*Gastrodia*, *Epipogium*, *Danxiaorchis*) in Orchidaceae [14–18]. Additional evidence of gene evolution stems from large datasets of protein coding gene sequences from Ericaceae and the remaining six monocot families (Thismiaceae, Burmanniaceae, Triuridaceae, Corsiaceae, Iridaceae, Petrosaviaceae) including mycoheterotrophic species [19, 20].

Despite being incomplete, the picture of mitogenome evolution in mycoheterotrophic plants emerging so far confirms the expectation of limited influence of the lifestyle. Size variation, structural diversity, and gene content of the mitogenome is comparable to that in other flowering plants. In some lineages, e.g., *Epirixanthes*, Thismiaceae and some orchids, a moderate s​ubstitution rate elevation has been found in mycoheterotrophic species compared to related autotrophs, but in other lineages, e.g., Ericales, no such rate elevation was found [12, 13, 20]. Mitogenomes of flowering plants often include foreign DNA either resulting from intracellular gene transfer (IGT) or horizontal gene transfer (HGT) [21]. IGT most frequently involves transfer from plastids to mitochondria (MTPTs), however, in mycoheterotrophic plants with reduced plastomes, the possibility of such transfer is reduced [16, 22]. In contrast to haustorial parasites, where the intimate contact between the plants and their hosts appear to promote HGT, the mycoheterotrophic lifestyle seems to have an opposite effect [8, 13, 23, 24]. In orchids, ancient events of HGT are observed from pathogenic fungi, but transfer between mycoheterotrophic plants and their mycorrhizal partners is not known [16, 25, 26]. Evidence of HGT between mycoheterotrophs and other angiosperms is scarce and also restricted to orchids [16, 18].

In order to increase our knowledge about mitogenome evolution in mycoheterotrophic plants, we here provide evidence from a lineage, Thismiaceae, where complete mitogenomes have not previously been sequenced. In order to explore potential intracellular transfer between the plastome and the mitogenome, the former is also sequenced and described. For the mitogenome we compare its characteristics with those of other mycoheterotrophs. In particular, we focus on foreign DNA and provide new evidence of potential HGT of plastid DNA from other angiosperms into the mitogenome of *Thismia*.

## Materials and methods

### Plant material and sequencing

Plant material of *Thismia angustimitra* Chantanaorr. was collected at the type locality, Phu Wua Wildlife Sanctuary, Buengkan Province, Thailand in 2012 and a voucher (N. Suphuntee & al. 734) is stored in the Forest Herbarium (BKF) in Bangkok. A silica gel preserved sample is stored at the Natural History Museum of Denmark under accession number C2848.

The silica gel preserved sample was used for total genomic DNA extraction as described by Petersen et al. [13]. Further preparations, including DNA shearing and library construction, prior to sequencing also followed the procedure previously described [13]. Sequencing of 100 bp paired-end fragments was performed on an Illumina Hiseq 2500 platform. Raw sequence reads were trimmed using Adapted Removal [27].

### Plastome assembly and annotation

The plastome of *T. angustimitra* was assembled using the plastome of *T. tentaculata* [6] as a reference. Cleaned sequence reads were mapped to the reference in Geneious v. 11.0.2 (Biomatters Ltd.) using the Map to Reference option under default settings. Consensus sequences were calculated based on the mapped reads and extracted. The extracted fragments were used for *de-novo* assembly using the default option in Geneious, and a few new rounds of mapping to reference, consensus extraction and *de-novo* assembly were applied to complete a circular plastome. Sequence annotation was also performed in Geneious v. 11.0.2 initially using *T. tentaculata* as a reference and adjusted following the availability of additional *Thismia* plastomes [5].

### Mitogenome assembly, annotation, and analyses of repeats and RNA editing

The mitogenome of *T. angustimitra* was assembled using GetOrganelle v. 1.7.7.1 [28] with the parameters “-k 21,45,65,85 -P 1000000 -F embplant_mt”, and the assemblies were visualized using Bandage v. 0.8.1 [29]. Contigs lacking genes and not initially assigned to the mitogenome pool were evaluated based on sequence depth and by blasting against NCBI GenBank. If the best matches were to other plant mitogenome sequences they were accepted as mitochondrial. R v. 4.5.2 [30] was used to plot average read depth of each contig against contig length and to calculate correlation statistics.

The Geneious Prime v. 2025.1.2 repeat finder was used to detect repeats in the mitogenome with a minimum size of 80 bp and a maximum of 5% mismatches. In order to test if the identified repeats were involved in repeat-mediated recombination, sequences having the alternative configuration around the repeats were constructed and used for mapping. Due to the short length of sequence reads, just 100 bp of sequence flanking the repeats was used. Paired sequence reads were mapped using high stringency, custom settings (0% mismatches allowed) in Geneious Prime v. 2025.1.2.

All mitogenome contigs were circular and due to limited existence of repeats (see below), only two chromosomes could potentially exist in different configurations. Gene annotation was done in Geneious Prime v2025.1.2 using primarily the potentially complete mitogenome of *Trichopus zeylanicus* (Dioscoreaceae) [31] and 36 complete or partial gene sequences from *Thismia rodwayi* [20] as references. For detection of tRNA genes tRNAscan-SE 2.0 [32, 33] was used.

To predict RNA editing in the protein coding genes the online version of Deepred-Mt [34] was applied with a minimum threshold for accepting a site as edited set to 0.9. For comparison of editing between autotrophic and mycoheterotrophic species of Dioscoreales, numbers of predicted edited sites were estimated for *T. angustimitra* and *Trichopus zeylanicus*. Published data from *Dioscorea oppositifolia* [35] were included in the comparison after verification of the numbers shown [35: p.11, Fig. 7] being estimated from Deepred-Mt using the same threshold as above.

### Analyses of intracellular and horizontal gene transfer

Nucleotide similarity between protein coding genes of *Thismia angustimitra* and *T. rodwayi* was calculated from pairwise alignments in Geneious Prime v. 2025.1.2 using the Translation Align option of Muscle v. 5.1 [36]. To detect potential foreign origin of the genes, they were all subjected to BLASTn [37] similarity searches against land plant mitochondrial sequences in NCBI GenBank. If the best matching taxa other than *Thismia* spp. were members of Dioscoreales, sequences were assumed to be native.

To detect recent intracellular sequence transfer from the plastid to the mitochondrial genome the plastome of *T. angustimitra* was blasted against each of the mitochondrial chromosomes using Geneious Prime 2025.1.2. To detect potentially more ancient transfers or sequences stemming from HGT all intergenic sequences from the mitogenome were subjected to BLASTn [37] similarity searches against complete chloroplast genomes in NCBI GenBank. Best matching sequences globally and from Dioscoreales were recorded for inserts exceeding 80 bp. For each detected insert, the Alien Index (AI) was calculated based on BLAST E-values [38]. The index measures how many orders of magnitude the scores from Dioscoreales and other angiosperms differ. A positive AI suggests a foreign nature of an insert and a negative AI a native nature. However, in the original publication AI values in the range 0 < AI < 45 were considered indeterminate [38]. The longest found insert, a 797 bp fraction of *psaA*, was subjected to phylogenetic analysis to confirm the origin (Anacardiaceae, Sapindales) suggested by similarity. Thirtythree full-length *psaA* sequences were downloaded from NCBI GenBank (Additional file 1). The sequences were chosen to span the entire angiosperm phylogeny and with denser sampling within Dioscoreales and Sapindales. All sequences, including the *Thismia psaA* fragment, were aligned using MAFFT v. 7.490 [39, 40] under default settings as implemented in Geneious Prime v. 2025.1.2. A maximum likelihood phylogenetic analysis with automated model selection [41] was performed using IQ-TREE v.3.1.4 [42] with ten runs, 10,000 ultrafast bootstrap (UB) replicates and using *Amborella* as the outgroup.

Since the detected sequences of plastome origin suggested that HGT might best explain the origin of some of them, the regions were all subjected to BLASTn [37] similarity searches against the entire nucleotide sequence database in NCBI GenBank. This approach was adopted to possibly detect whether inserts originated directly from plastid sequence or if they could be the results of prior plastid to mitogenome intracellular transfer in donor species followed by HGT of mitogenome sequence. Additionally, even the entire intergenic sequence space of *Thismia angustimitra* was subjected to BLASTn [37] similarity searches against the entire nucleotide sequence database in NCBI GenBank. Best results were recorded for sequences exceeding 200 bp.

Potential HGT from fungi in particular was explored using the same approach as applied by Sinn and Barrett [25]. This targeted approach could potentially find sequences missed by the above search approach. Using custom BLASTn [37] as implemented in Geneious Prime v. 2025.1.2 all mitogenome chromosomes were screened for presence of sequences similar to the mitogenome of *Ustilago maydis* (DQ157700) representing potential pathogenic fungi and *Rhizophagus irregularis* (NC014489) representing potential host fungi. The parameters of BLASTn were set to: word size 7, scoring match/mismatch 2/−3, gap opening/extension 3/3, and E threshold 1e^− 5^.

### Phylogenetic analysis

To place the accession of *Thismia angustimitra* in a phylogenetic context, a previously assembled dataset including 42 species of *Thismia* and 12 other representatives of Dioscoreales was used [43]. The dataset includes sequences from the mitochondrial *atp1* gene and the nuclear 18 S and ITS regions (ITS1-5.8 S-ITS2). Thus, these nuclear regions were extracted from the *T. mirabilis* sequencing data using sequences from the dataset as reference. To insert the new sequences into the matrix each locus was realigned using default settings of MAFFT v. 7.490 [39, 40] as implemented in Geneious Prime v. 2025.1.2. Dubiously aligned positions, in particular in the ITS regions, were pruned using GBlocks v. 0.91b [44] with default parameters, but allowing gap positions in the final blocks and allowing less strict flanking positions. Alignment and pruning methods were similar to those used by Shepeleva et al. [43]. Phylogenetic analysis was performed on the concatenated alignment using RAxML v. 8.2.11 [45] as implemented in Geneious Prime v. 2025.1.2. The settings were also chosen to mimic Shepeleva et al. [43] as close as possible, thus the nucleotide model was set to GTR+Gamma and the algorithm to rapid bootstrapping and search for best-scoring ML tree running 100 bootstrap replicates with data being partitioned corresponding to the three loci and using *Dioscorea* as the outgroup.

## Results

Sequencing resulted in 66,750,876 raw paired-end reads. After trimming 61,734,321 reads remained for subsequent analysis.

### Characteristics of the plastome and the mitogenome

**Fig. 2:**
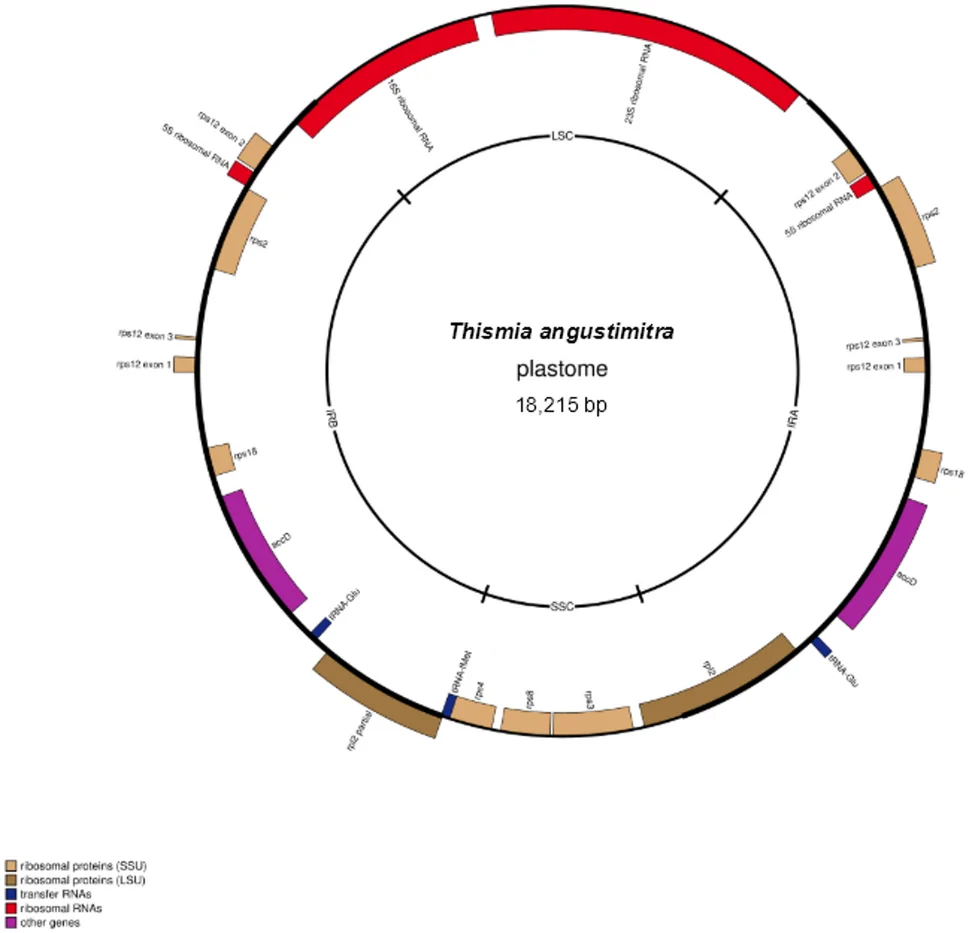
The plastome of *Thismia angustimitra*. The direction of transcription of genes drawn inside the circle is clockwise and counterclockwise for those drawn outside. Drawing made using OGDRAW v. 1.3.1 [46]

The plastome was assembled into a 18,215 bp circular molecule with a mean sequence read depth of 1,248× (Fig. 2). It has a standard quadripartite structure including an inverted repeat of 5,876 bp. The content of genes is low including eight protein coding genes, three rRNA genes and two tRNA genes (Table 1). All three exons of the gene *rps12* are present, but the intron between exon 2 end exon 3 is unusually long and includes complete *rrn5* and *rps2* genes.

**Table 1:** Gene content of the plastome of *Thismia angustimitra*

Gene group	Genes
Acetyl-CoA carboxylase	*accD* (×2)
Ribosomal proteins (LSU)	*rpl2*
Ribosomal proteins (SSU)	*rps2* (×2), *rps3*, *rps4*, *rps8*, *rps12*, *rps18* (×2)
Ribosomal RNAs	*rrn5* (×2), *rrn16*, *rrn23*
Transfer RNAs	*trnE-UUC* (×2), *trnfM-CAU*

**Fig. 3:**
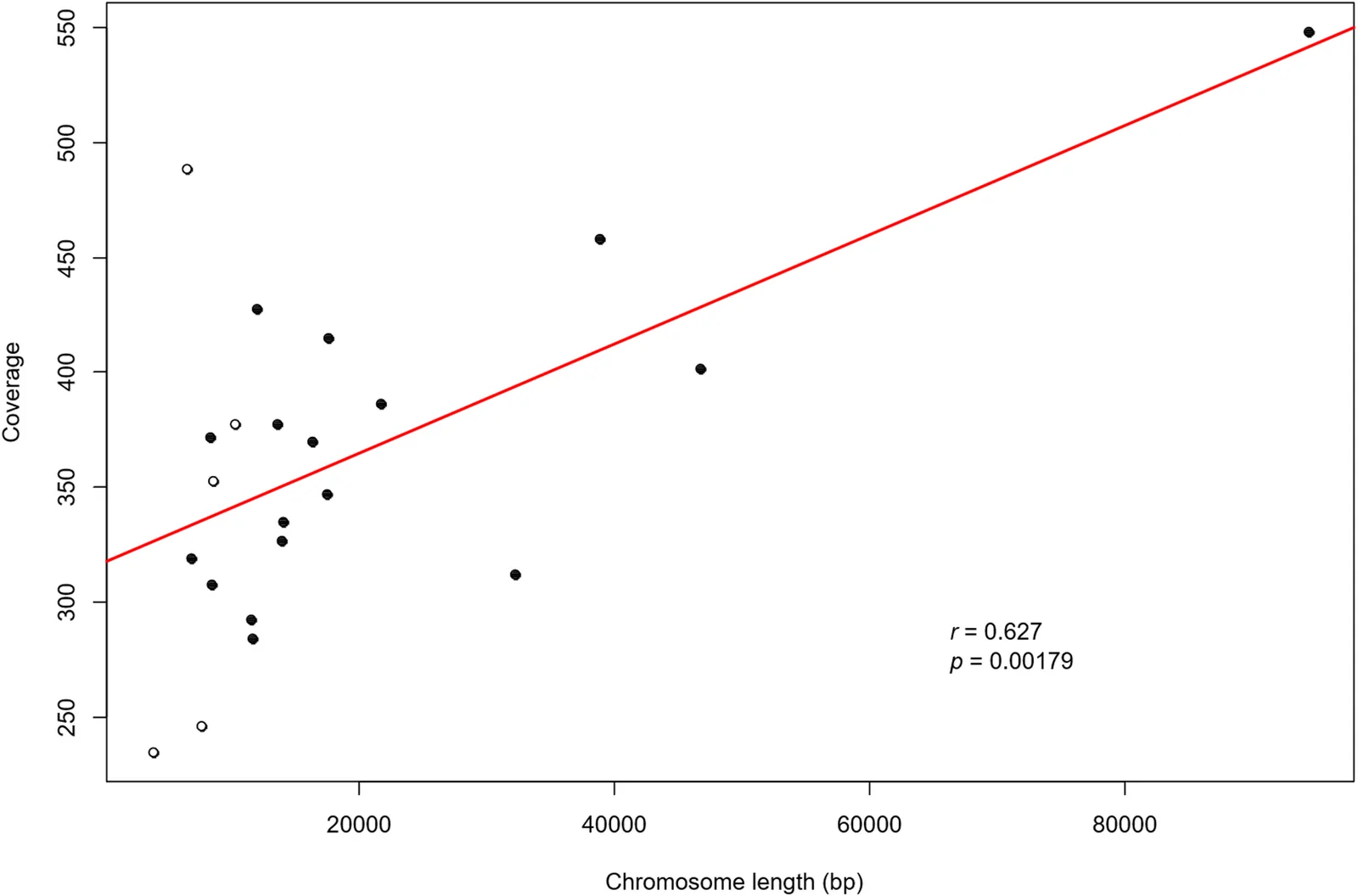
Linear regression illustrating the positive correlation between mean sequence coverage and chromosome length. Chromosomes with and without genes are shown as filled and open circles, respectively

The mitogenome of *T. angustimitra* was assembled into 22 circular chromosomes with a total length of 422,400 bp (Table 2, Additional file 2). The chromosomes range in size from 3,871 bp to 94,341 bp. Five chromosomes contain no genes, but show sequence similarity to other non-coding plant mitochondrial sequences and read mapping shows that they have a read depth (ca. 235–489×) largely within the range of the chromosomes including genes (ca. 284–548×). The GC content of the geneless chromosomes (41.2–43.1%) is also similar to that of the chromosomes including one or more genes (41.6–46%) supporting their mitochondrial origin. Average read depth is moderately correlated with chromosome length (*r* = 0.627, *p* < 0.01) with longer chromosomes having higher coverage, but there is no significant correlation between the sets of chromosomes with or without genes (*t* = 0.6983, *p* = 0.5756) (Fig. 3).

**Table 2:** Characteristics of the mitochondrial chromosomes of *Thismia angustimitra*

Chromosome	Length (bp)	Read depth	GC %	Gene content
Chrom 1	94,341	548.1	44.1	*ccmFn*, *cox3*, *matR*, nad1^*^ exon 2–3, *nad4*, nad5^*^ exon 4–5, *nad7*, *rps2*, *rrn26*, *sdh4*, *trnP-UGG*
Chrom 2	46,704	401.4	43.4	nad2^*^ exon 1–2, *trnD-GUC*,* trnL-CAA*
Chrom 3	38,857	458.2	43.4	*atp1*, *atp4*, *atp9*, *nad3*, *nad4L*, *rpl2*, *rpl16*, *rps3*, *rps10*, *rps12*, *rps19*, *trnfM-CAU*
Chrom 4	32,244	312.2	42.8	*atp6*, *ccmFc*, *cob*, *rpl5* Ψ, *rps11*, *rps14*
Chrom 5	21,669	386.3	43.4	*cox1*
Chrom 6	17,561	414.6	41.8	nad1^*^ exon 5, nad5^*^ exon 3, *rps4*
Chrom 7	17,491	346.6	43.2	*ccmC*, *cox2*, *rps13*
Chrom 8	16,334	369.9	43.2	*trnK-UUU*
Chrom 9	14,026	335.1	43.3	nad1^*^ exon 1
Chrom 10	13,935	326.5	42.2	nad1^*^ exon 4
Chrom 11	13,582	377.1	43.8	*ccmB*, nad2^*^ exon 3–5, *nad9*
Chrom 12	11,980	427.3	43.2	*rps7*, *rrn5*, *rrn18*
Chrom 13	11,659	284	41.6	*trnF-AAA*, *trnP-UGG*
Chrom 14	11,540	292.1	43.5	*mttB*
Chrom 15	10,238	377.1	41.6	
Chrom 16	8,584	352.3	41.3	
Chrom 17	8,447	307.6	43.6	*nad6*
Chrom 18	8,352	371.7	46	nad5^*^ exon 1–2
Chrom 19	7,688	245.8	41.3	
Chrom 20	6,830	319.1	44	*atp8*, *rps1*
Chrom 21	6,467	488.6	43.1	
Chrom 22	3,871	234.6	41.2	

^*^ trans-spliced gene

The gene complement includes 39 protein coding genes, three rRNA genes and six tRNA genes (Tables 2 and 3). One of the protein coding genes (*rpl5*) is a pseudogene. Three genes are trans-spliced (*nad1*, *nad2*, *nad5*) with exons distributed on two or three different chromosomes.

**Table 3:** Gene content of the mitogenome of *Thismia angustimitra*

Gene group	Genes
ATP synthase	*atp1*, *atp4*, *atp6*, *atp8*, *atp9*
NAD(P)H dehydrogynase	*nad1*, *nad2*, *nad3*, *nad4*, *nad4L*, *nad5*, *nad6*, *nad7*, *nad9*
Cytochrome B	*cob*
Cytochrome c biogenesis	*ccmB*, *ccmC*, *ccmFc*, *ccmFn*
Cytochrome c oxidase	*cox1*, *cox2*, *cox3*
Maturase	*matR*
Membrane transport protein	*mttB*
Ribosomal proteins (LSU)	*rpl2*, *rpl5* Ψ, *rpl16*
Ribosomal proteins (SSU)	*rps1*, *rps2*, *rps3*, *rps4*, *rps7*, *rps10*, *rps11*, *rps12*, *rps13*, *rps14*, *rps19*
Succinate dehydrogenase	*sdh4*
Ribosomal RNAs	*rrn5*, *rrn18*, *rrn26*
Transfer RNAs	*trnF-AAA*,* trnD-GUC*, *trnfM-CAU*, *trnK-UUU*, *trnL-*CAA, *trnP-UGG* (×2)

The mitogenome contains few repeats (Table 4) accounting for just 0.8% of the total genome size, and repeat-mediated recombination appears limited. Chromosome 4 includes a 498 bp direct repeat (Repeat 1), and it can be broken up in two smaller circular chromosomes of 14,443 bp and 17,801 bp, respectively. Due to the short sequence reads and insert size even the paired reads cannot be used to reveal which configurations are present. The only repeat that appears to be involved in recombination is the 99 bp long Repeat 8 located in an intergenic region of chromosome 1 and within the intron of *rps3* in chromosome 3. The presented configuration is supported by 79 (chrom 1) and 80 (chrom 3) read pairs, while the alternative configuration splitting *rps3* is supported by just one (chrom 1/chrom 3) and 13 (chrom 3/chrom 1) read pairs. Thus, a total of 8.6% reads supports the alternative configurations. For all other repeats there are no paired reads supporting alternative configurations.

**Table 4:** Repeats (> 80 bp, < 5% mismatch) in the mitogenome of *Thismia angustimitra*

Repeat no.	Length (bp)	Sequence identity (%)	Occurrences	Location and orientation
Repeat 1	498	100	2	Chrom 4 (2×, direct)
Repeat 2	180	100	2	Chrom 4, Chrom 7 (partially overlapping repeat 1)
Repeat 3	158	100	3	Chrom 4 (2×, direct), Chrom 7 (part of repeats 1, 2)
Repeat 4	138	99	2	Chrom 2, Chrom 16
Repeat 5	127	100	2	Chrom 1, direct (one copy in *nad7* intron 4)
Repeat 6	123	100	2	Chrom 5, Chrom 6
Repeat 7	116	96	2	Chrom 6, Chrom 14
Repeat 8	99	100	3	Chrom 1 (2×, direct, part of repeat 4), Chrom 3 (in *rps3* intron)
Repeat 9	98	99	2	Chrom 1, Chrom 17
Repeat 10	88	100	2	Chrom 5, Chrom 22

### RNA editing and foreign DNA

RNA editing was predicted for all 38 potentially functional protein coding genes in *T. angustimitra* and in complete genes available in the mitogenome of *Trichopus zeylanica* (Table 5). In the latter some core genes are missing either completely (e.g., *cox3*, *nad7*) or partially (e.g., individual exons of *nad1*, *nad2*, *nad5*) suggesting that the mitogenome may not be complete. The gene complement of *Dioscorea oppositifolia* is complete and includes 612 edited sites compared to just 499 in *Thismia angustimitra*. For 27 genes shared between all three species, the total number of edited sites is lower in *Thismia* (326 sites) compared to both *Dioscorea* (476 sites) and *Trichopus* (385 sites). In *Thismia*, no clusters of C-to-T substitutions potentially indicating retroprocession were observed (data not shown).

**Table 5:** Number of predicted edited sites in mitochondrial genes of *Thismia angustimitra*, *Dioscorea oppositifolia* [35], and *Trichopus zeylanicus* [31]

Gene	Number of edited sites Thismia Dioscorea Trichopus			
	Thismia	Dioscorea	Trichopus	
*atp1*	7	11	5	
*atp4*	10	13	13	
*atp6*	14	24	20	
*atp8*	5	5	4	
*atp9*	4	4	9	
*ccmB*	27	38	34	
*ccmC*	28	39	39	
*ccmFc*	12	6	12	
*ccmFn*	30	32	--	
*cob*	11	17	15	
*cox1*	18	26	22	
*cox2*	14	16	17	
*cox3*	14	12	--	
*matR*	8	14	--	
*mttB*	27	36	22	
*nad1*	26	29	--	
*nad2*	29	34	--	
*nad3*	17	17	16	
*nad4*	45	54	55	
*nad4L*	9	12	11	
*nad5*	24	33	--	
*nad6*	13	16	17	
*nad7*	35	37	--	
*nad9*	12	14	13	
*rpl2*	1	0	--	
*rpl16*	9	10	14	
*rps1*	3	3	--	
*rps2*	5	4	3	
*rps3*	5	12	10	
*rps4*	​15	13	13	
*rps7*	1	2	2	
*rps10*	2	4	0	
*rps11*	1	2	--	
*rps12*	7	8	8	
*rps13*	5	6	4	
*rps14*	2	2	2	
*rps19*	2	4	5	
*sdh4*	2	3	--	
All genes	499(326)	612(476)	385	

Numbers in parentheses are sums of edited sites in genes in common for all three species

Nucleotide similarity of protein coding genes between *T. angustimitra* and *T. rodwayi* is very high ranging from 91.9% (*nad9*) to 100% (*nad4L*) with an average of 97% (Table 6), and all gene sequences have best BLASTn matches with other sequences from Dioscoreales.

**Table 6:** Nucleotide similarity between protein coding genes of *Thismia angustimitra* and *T. rodwayi* [20]

Gene	Length (bp) T. angustimitra	Length (bp) T. rodwayi	Nucleotide identity (%)
*atp1*	1524	1492^1^	98.9
*atp4*	579	479^1^	98.1
*atp6*	810	705^1^	96.7
*atp8*	477	455^1^	94.5
*atp9*	225	223^1^	99.6
*ccmB*	624	619^1^	96.8
*ccmC*	717	689^1^	98.7
*ccmFc*	1320	974^1^	94.1
*ccmFn*	1716	1444^1^	92.6
*cob*	1188	1171^1^	96.9
*cox1*	1620	1578	97.2
*cox2*	774	632^1^	97.5
*cox3*	798	798	98.6
*matR*	1971	1878^1^	95.5
*mttB*	825	696^1^	98.0
*nad1*	981	978^1^	99.0
*nad2*	1569	1448^1^	96.2
*nad3*	357	351^1^	98.9
*nad4*	1482	1467^1^	97.8
*nad4L*	303	303	100
*nad5*	1995	1962^1^	97.9
*nad6*	537	513^1^	99.2
*nad7*	1185	1185	99.0
*nad9*	561	555	91.9
*rpl2*	1527	1296^1^	96.9
*rpl5*	Ψ	570^1^	n.a.
*rpl16*	516	516	99.0
*rps1*	591	480^1^	98.8
*rps2*	600	560^1^	95.2
*rps3*	1644	1585^1^	94.3
*rps4*	1038	997^1^	93.7
*rps7*	447	441^1^	97.1
*rps10*	336	n.a.	n.a.
*rps11*	528	n.a.	n.a.
*rps12*	375	353^1^	99.7
*rps13*	345	345	95.8
*rps14*	303	n.a.	n.a.
*rps19*	288	224^1^	96.0

^1^Sequence not complete

**Fig. 4:**
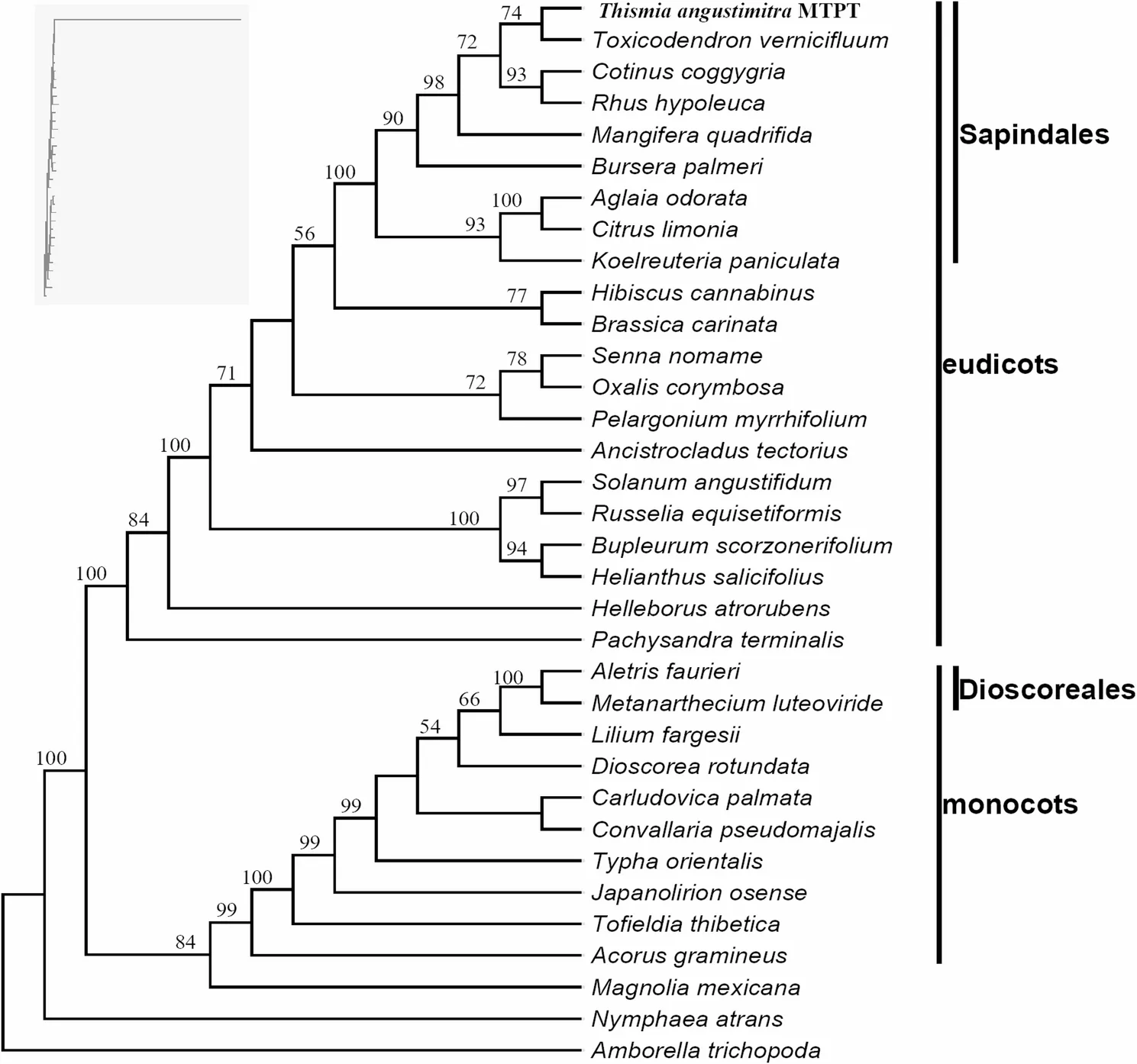
ML phylogeny based on plastid *psaA* sequences of selected angiosperms and a MTPT from *Thismia angustimitra*. Numbers on branches are ultrafast bootstrap support > 50%. Upper left insert shows the tree with proportional branch lengths

No similarity to fungal mitogenome sequences was detected, and the mitogenome contains little DNA of plastid origin (Table 7). Sixteen inserts, MTPTs, with a total length of 4,068 bp were detected spanning just 0.96% of the mitogenome. One insert was only found in *Thismia* and five have negative AI scores suggesting that they stem from intracellular transfer. However, only two of the latter have numerically high AI scores leaving the nature of the remaining three insert uncertain. A single insert was found only outside of Dioscoreales and another nine, many of which include sequences that are not present in the reduced *T. angustimitra* plastome, have positive AI scores suggesting a foreign nature. However, only two that are both most similar to members of Anacardiaceae have scores > 45. Phylogenetic analysis of the longest MTPT (a fragment of *psaA*) with an AI score of 46 resulted in a tree with the MTPT placed on a very long branch (Fig. 4). Still its position within Sapindales is strongly supported (100% UB) as is the position within Anacardiaceae (98% UB). For seven of the MTPTs similar sequences were found in different eudicot mitogenomes (Table 7). The difference in similarity between MTPTs in *T. angustimitra* and plastid versus mitochondrial sequences is mostly negligible except for a 569 bp fragment in chromosome 1 showing substantially higher similarity to the mitogenome sequence (87.9%) of *Diospyros* (Ebenaceae) than to the plastid sequence (73.8%) of *Liriodendron* (Magnoliaceae).

**Table 7:** Sequences of plastid origin located in the mitogenome of *Thismia angustimitra*

MT location	Length (bp)	Best match non-Dioscoreales CP Taxon^1^ similarity (%) E-value			Best match Dioscoreales CP Taxon similarity (%) E-value			AI	Best match MT^2^ Taxon Similarity (%) E-value			AI	CP region
Chrom 1: 19,399 − 19,502	104	none			*Thismia* (self)	77.7	6e-15		none				*rrn5*
Chrom 1: 38,245 − 38,813	569	*Liriodendron*	73.8	2e-107	*Aletris*	69.5	4e-71	38	*Diospyros*	87.9	0.0	38	*ndhJ-ndhK*
Chrom 1: 55,947 − 56,743^3^	797	*Toxicodendron*	71.7	2e-110	*Aletris*	68.2	9e-68	46	none				*psaA*
Chrom 1: 56,792 − 57,154^3^	363	*Rhus*	70.9	7e-48	*Aletris*	78.1	1e-11	83	none				*ycf3*
Chrom 1: 57,362 − 57,513	152	*Peperomia*	86.2	2e-42	*Tacca*	84.9	1e-45	-8	none				*rpl23*
Chrom 4: 3,114-3,354^3^	241	*Bouea*	72.8	7e-26	*Dioscorea*	71.5	5e-19	16	*Mangifera*	72.8	3e-25	14	*psaA*
Chrom 4: 3,406-3,547	142	*Pseudoscourfieldia*	73.8	4e-17	*Thismia* (self)	85.5	2e-36	-44	none				*rrn16*
Chrom 4: 7,398-7,532	135	*Epifagus*	73.5	6e-21	*Thismia* (self)	71.9	2e-14	15	none				*rrn16*
Chrom 6: 5,515-5,678	164	*Medicago*	80.0	6e-25	*Dioscorea*	78.4	3e-26	-3	none				*rrn23*
Chrom 6: 9,296-9,409	114	Euphorbiaceae	91.6	9e-39	*Metanarthecium*	86.6	3e-37	4	*Striga*	91.6	9e-39	4	*atpA*
Chrom 7: 134–325	192	*Croton*	75.4	7e-24	none				*Sapindus*	75.0	7e-25		intergenic
Chrom 9: 13,882 − 115	260	*Rapatea*	70.8	7e-31	*Thismia* (self)	77.7	3e-56	-58	none				*accD*
Chrom 10: 3,618-3,913^3^	296	*Incarvillea*	73.9	4e-37	*Aletris*	72.7	1e-35	3	*Neobartsia*	74.6	4e-37	3	*psaB*/*psaA*
Chrom 16: 3,560-3,647	88	*Danaea*	83.8	3e-11	*Thismia* (self)	81.8	7e-13	-4	none				*rrn16*
Chrom 18: 1,916-2,189	274	*Semecarpus*	68.7	3e-24	*Dioscorea*	71.7	2e-12	27	*Mangifera*	68.7	1e-17	12	*rpoB*
Chrom 19: 5,603-5,779^3^	177	*Rubroshorea*	73.6	6e-25	*Burmannia*	69.2	2e-16	20	*Salvia*	75.0	1e-25	21	*psaB*

1) Typically, many matches, sometimes from distantly related taxa, have very similar scores. Only the top-scoring taxon listed

2) Some sequence regions also match mitochondrial sequences from diverse angiosperms

3) Possibly parts of a single, larger, original transfer

By searching for similarity between mitochondrial intergenic sequences and all NCBI GenBank nucleotide sequences 155 mitogenome regions ranging in length from 201 bp to 3,614 bp were recorded (Additional file 4). In total they cover 87,450 bp, i.e., ca. 21% of the entire genome or ca. 25% of the intergenic sequence. The sequences are distributed on all chromosomes, but not evenly with coverage varying from 12% (chromosomes 7, 14 and 17) to 41% (chromosome 21). With a single exception all best sequence matches are mitochondrial intergenic sequence with 67–100% similarity scores. The one exception is a 789 bp sequence on chromosome 12 with low similarity (71%) to an uncharacterized nuclear protein sequence from *Primulina* (Gesneriaceae). Of the remaining 154 sequences, only nine (ca. 1% of the intergenic sequence) have best similarity to other mitochondrial sequences from Dioscoreales. Another 21 (ca. 3% of the intergenic sequence) are most similar to other monocot mitochondrial sequences, five are most similar to Amborellaceae or Lauraceae, but the majority of the sequences (119) are most similar to a wide range of eudicots (31 families in 16 orders). Most families are recorded with a best match only once or twice, but two families stand out with 26 and 19 sequences having their best match to Malvaceae and Orobanchaceae, respectively. Sapindales, as mentioned above, is represented by 19 sequences; six of them best matching Anacardiaceae, 11 best matching Sapindaceae.

In most cases there is no clear pattern between the taxonomic identity of best matching sequences and the location within the mitogenome of *Thismia angustimitra* (Additional file 4). However, a few exceptions can be mentioned: in chromosome 1 three closely located sequences covering 1,962 bp of a 4,116 bp region have their best match to three widely spread regions in *Sapindus mukorosii* (Sapindaceae), in chromosome 5 four closely located sequences covering 2,389 bp of a 4,387 bp region match widely spread regions in *Pityranthe trichosperma* (Malvaceae), and also in chromosome 5 four regions covering 2,847 bp of a 3,098 bp region match three widely spread regions in *Aeginetia indica* (Orobanchaceae). Further, among the small geneless chromosomes, chromosome 21 includes five sequences (41% of the chromosome), which all have their best match to species of Malvaceae. Finally, in the larger chromosome 2, 13 of 18 identified sequence regions have best matches to species of Orobanchaceae (*Christisonia kwangtungensis*, *Aeginetia indica* and *Alectra orobanchoides*). These regions include the longest (up to 3,614 bp) matching sequences and mostly with very high sequence similarity (89–100%), but synteny between sequence regions is limited.

### Phylogeny

**Fig. 5:**
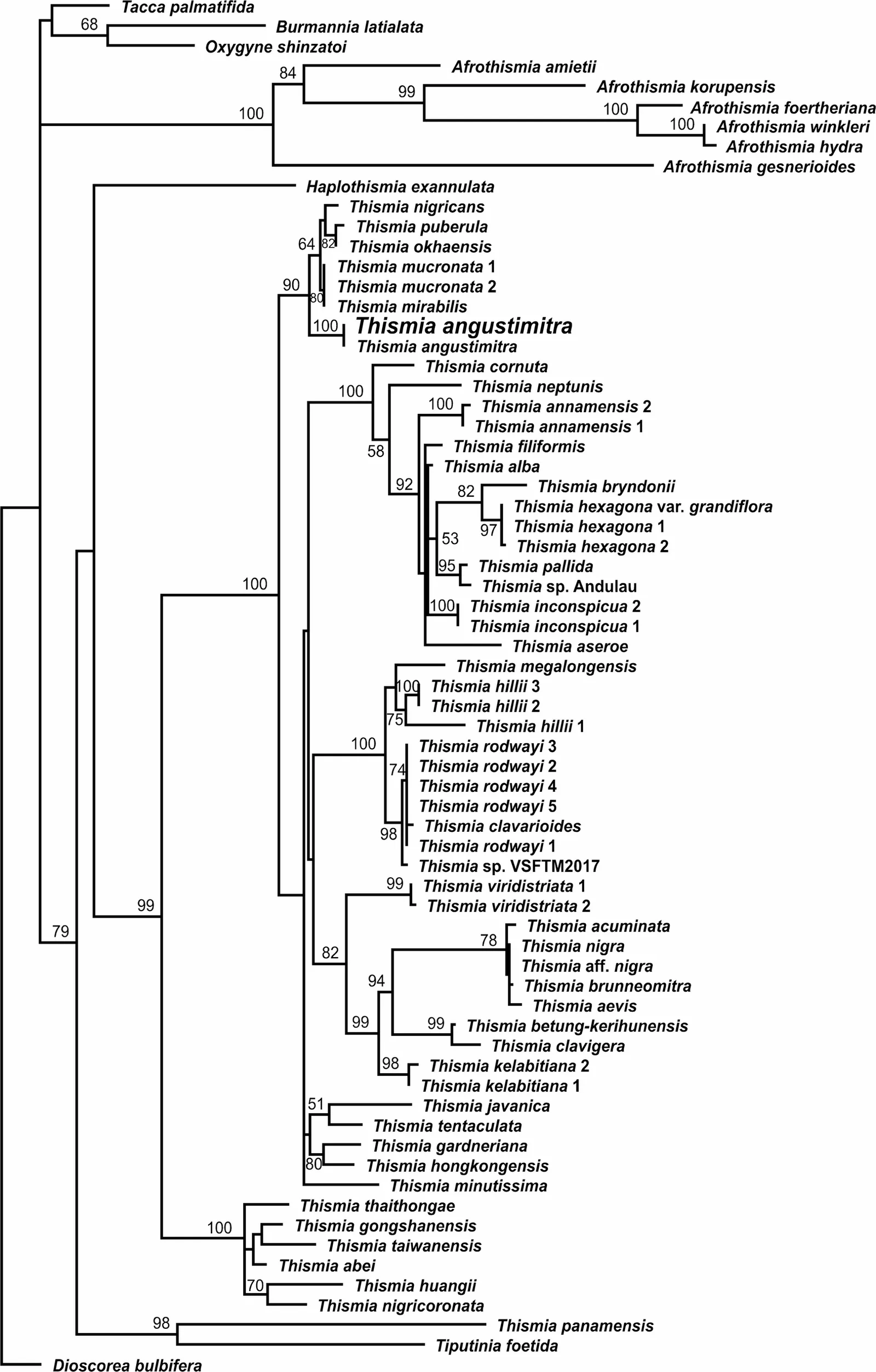
Phylogeny of *Thismia* highlighting the position of *T. angustimitra*. The tree is based on a maximum likelihood analysis of three loci: mitochondrial *atp1*, nuclear 18 S and ITS (ITS1-5.8 S-ITS2). Numbers on branches are bootstrap support > 50%

The phylogenetic analysis of *Thismia* placed the added specimen with 100% bootstrap support as the sister to another specimen of *T. angustimitra* despite of low support for many other clades in the tree (Fig. 5). *Thismia angustimitra* is placed in a well-supported clade (90%) including *T. mirabilis*, *T. mucronata*, *T. okhaensis*, *T. puberula*, and *T. nigricans*, but a sister group relationship of *T. angustimitra* to the remaining species is weakly supported (64%).

## Discussion

In a previous phylogenetic analysis of *Thismia* based on three loci (mitochondrial *atp1*, nuclear ITS and 18 S), *T. angustimitra* was represented only by ITS data [43]. Here, sequence data from all three loci are included and as expected, the phylogenetic analysis firmly group the two specimens of *T. angustimitra* together. A close relationship of *T. angustimitra* to a group of five other species from Thailand and Vietnam is also confirmed.

### Plastome characteristics

Plastomes of fully mycoheterotrophic plants are usually strongly degenerate [2], and the present is no exception. Several plastomes have previously been sequenced from other species of *Thismia*, and the currently sequenced plastome from *T. angustimitra* falls within the described range of variability with regards to total length, IR length, and gene composition [4–7]. The few genes hitherto found in all completely sequenced plastomes (*accD*, *rpl2*, *rps12*, *rrn16*, *rrn23*, *trnE-UUC*) are also present in *T. angustimitra*. Of the additional eight ribosomal genes that have been found in one or more species, five are present and potentially functional in *T. angustimitra* (*rps2*, *rps3*, *rps4*, *rps8*, *rps18*) and three are absent (*rpl16*, *rps11*, *rps19*). Compared to a previously sequenced partial plastome from another specimen of *T. angustimitra* (S. Chantanaorrapint & C. Promma 3904) [4], the present sequence includes a complete *rps3* gene in the region, where the other specimen was incomplete. Based on the evolutionary scenario of gene loss described by Garrett et al. [5] presence of *rps3* was expected. Except for a single base pair difference in *rrn16* the two samples, that have been collected at the same locality are identical. The slightly shorter plastome length of 18,204 bp indicated by Garrett et al. [5] for the S. Chantanaorrapint & C. Promma 3904 specimen stems from the sequence in GenBank (MT943130), which includes a long string of N’s inserted as an estimate of missing sequence.

While pseudogenization and gene loss appear to be an ongoing process in Thismiaceae, a previous study found that most protein coding genes remain under purifying selection with only occasional and rarely significant relaxation of selection [5]. Therefore, it was suggested that relaxation of selection, pseudogenization and gene loss may happen within just a short evolutionary time frame [5]. This hypothesis could be consistent with functional transfer of remaining plastid, housekeeping genes to the nuclear genome. Only after transfer could the plastid gene copy degrade. Currently, Thismiaceae lacks nuclear genome data to test this extended hypothesis.

### Mitogenome characteristics

In contrast to the strongly modified plastomes of fully mycoheterotrophic plants, their mitogenomes are far less affected by the lifestyle. However, based on the sequence of the complete nuclear genome of *Gastrodia elata* showing expansion of genes involved in some metabolic processes, Yuan et al. [15] suggested that the relatively large mitogenome (1,339 kb) also found in the species supported a hypothesis of mitogenome and gene expansion being related to the mycoheterotrophic lifestyle. However, later evidence does not generally show expanded mitogenomes in mycoheterotrophic plants. While several species of *Gastrodia* have mitogenomes exceeding 1 mb in size and one even exceeding 2 mb, others have standard sizes around 5 − 600 kb [16], and other mycoheterotrophic orchids have even smaller mitogenomes (398 − 417 kb) [14, 17]. Neither is expansion of mitogenomes observed in the eudicots *Epirixanthes* (365 kb [13]) and *Monotropa* (810 kb [12]), and the present mitogenome of *Thismia* (422 kb) is smaller than mitogenomes of autotrophic members of Dioscoreales (*Trichopus* 709 kb [31], *Dioscorea* 493 kb [35]). In the Orobanchaceae, it was recently demonstrated that reduction in plastome size and function is correlated with expansion of the mitogenome [47]. A potential explanation could be that relaxed selection on the plastome affects nuclear encoded repair and maintenance mechanisms common to both organelles, leading to the proliferation of repeats in the mitogenome [47, 48]. The present data for *Thismia*, showing no mitogenome expansion and a limited repeat content, do not support this as a general trajectory of a heterotrophic or achlorophyllous lifestyle.

Due to the structural complexity and repetitive nature of many plant mitogenomes, assembly may be challenging and require long reads for trustworthy assembly. However, the mitogenome of *T. angustimitra* includes remarkably few repeats and the assembly was relatively unproblematic. The configuration including 22 circular chromosomes is not unusual compared to mitogenomes of other mycoheterotrophic plants or flowering plants in general. Despite pronounced structural variation and lability, at least partly caused by repeat-mediated recombination [49, 50], mitogenomes have often been depicted as single circular molecules, but evidence of multichromosomal mitogenomes is increasing [51]. This includes mycoheterotrophic plants and possibly orchids in particular, where 23 and 26 circular chromosomes have been found in species of *Epipogium*, and between 37 and 71 almost exclusively circular chromosomes have been found in species of *Gastrodia* [16–18]. In the mycoheterotrophic eudicots *Epirixanthes* and *Monotropa*, the mitogenomes have been depicted as one and two chromosomes, respectively, but in both cases the genome includes multiple repeats allowing for homologous recombination and existence of other structural variants [12, 13].

In multichromosomal mitogenomes differences in sequencing depth may be interpreted as due to unequal abundance of individual chromosomes [16, 52]. In *Gastrodia*, an average three-fold difference in intragenomic chromosomal copy number was detected, but with larger interspecific differences [16]. Here, as in the holoparasitic *Lophophytum* [52], we only observe a minor difference in intragenomic sequencing coverage, suggesting that all chromosomes occur at about the same frequency. As in previous studies [16, 53], sequence coverage is positively correlated with chromosome length, but in *Thismia* as well as in *Lophophytum* and *Silene* it appears not to be correlated with presence or absence of identifiable genes [52, 53]. Why chromosomes without genes are maintained in the mitogenome remains to be understood [52, 54]. Possibly, they contain yet unidentified sequence elements of importance for mitogenome function, or alternatively they represent a step towards deletion and streamlining of the mitogenome.

### Repeats and recombination in the mitogenome

A striking observation regarding the mitogenome of *T. angustimitra*, is the low abundance of sequence repeats and the extremely limited recombinational activity. As such, the mitogenome is comparable to those of holoparasitic *Thonningia sanguinea* and *Ombrophytum subterraneum* (Balanophoraceae) both characterized by multiple circular chromosomes, presence of some long and medium sized repeats, but little or no recombination between them [55, 56]. It was suggested that recombination could have been hindered by decreased similarity of repeats due to the accumulation of mutations, possibly caused by malfunctioning of nuclear-encoded organellar DNA replication, recombination and repair mechanisms [55]. Accumulation of mutations would also be more pronounced in taxa with increased substitution rates, such as many parasitic and mycoheterotrophic plants, including *Thismia* [20]. In a multichromosomal mitogenome of *Silene*, reduced recombinational activity was also found, and based on a general observation of occurrence of multichromosomal mitogenomes in lineages with accelerated substitution rates, it was suggested that the multichromosomal mitogenome structure might serve as a means of reducing mutational load [53]. Possibly, even by deleting entire chromosomes without genes.

### Intracellular and horizontal gene transfer

It is well known that mitogenomes of angiosperms can include large amounts of foreign DNA either stemming from IGT or from HGT [21]. In contrast to haustorial parasites, where the physical connection to host plants facilitates transmission of DNA or RNA, and hence increase the likelihood of HGT, no HGT has been found between mycoheterotrophs and their mycorrhizal, fungal partner [8, 25]. However, in orchids evidence of ancient HGT from pathogenic fungi exists [16, 25, 26]. With the lack of both mycorrhizal and pathogenic fungal DNA in *Thismia*, orchids remain the only mycoheterotrophic lineage whose mitogenomes have acquired fungal sequences through HGT.

In mycoheterotrophs, evidence of HGT from other land plants is extremely scarce and has also been confined to orchids. In *Gastrodia*, mitogenomes are proposed to house a few intergenic regions stemming from HGT from unidentified angiosperms, and in the chlorophyllous orchid *Nervilia* two gene copies were most likely of eudicot origin [16, 18]. In *Thismia angustimitra*, no extra mitochondrial gene copies were found, and all genes have a very high similarity to the genes in another species of *Thismia* as well as to other species of Dioscoreales, thus indicating a native nature. A previous phylogenetic analysis including several species of *Thismia* did not detect HGT either [20]. Further support for the native nature of *Thismia* genes comes from the pattern of predicted RNA edited sites. A lack of editing across an entire gene, or clusters of C-to-T changes within part of a gene, can indicate that RNA-mediated retroprocessing has replaced the whole or part of that gene [22, 57]. In parasitic Orobanchaceae, such replacements involve genes having been horizontally transferred [22]. In *Thismia*, however, comparison of edited sites with *Dioscorea* and *Trichopus* does not suggest any retroprocessing, despite a lower number of edited sites in *Thismia*. In Orobanchaceae, reduced RNA editing was hypothesized result from increased substitution rates, with both traits being correlated with the evolution of parasitism [22]. Although the level of RNA editing is lower in *Thismia angustimitra* than in *Dioscorea* and *Trichopus*, and a previous study has demonstrated an increased substitution rate in Thismiaceae [20], more data are needed to confirm a correlation between RNA editing and a mycoheterotrophic lifestyle.

In heterotrophic plants with reduced plastomes, conspecific intracellular transfer diminish, but their mitogenomes can still contain fragments of plastid DNA originating through HGT or ancient fragments of plastid origin having been vertically transmitted during evolutionary time [e.g., 13, 16, 22]. In *Thismia angustimitra*, the mitogenome has a very low content of MTPTs. While only a few short MTPTs most likely stem from conspecific intracellular transfer, other – some of which include sequence no longer present in the very reduced *Thismia* plastome – may stem either from more ancient intracellular transfer events or from HGT. Although differences in similarity for some MTPTs between sequences from Dioscoreales and those from other angiosperms are minor and the corresponding AI values equally inconclusive, the general pattern is that most MTPTs are more similar to plastome sequences of various eudicots and magnoliids than to those from Dioscoreales. This lends support to a hypothesis of HGT and thus contrasts with the pattern in *Epirixanthes*, where ancient intracellular transfer was thought to account for a large number of MTPTs [13]. Also in *Monotropa*, ancient intracellular transfer was proposed to account for MTPTs, but no data were presented to support the hypothesis [12]. In the mycoheterotrophic orchid *Epipogium*, a few MTPTs were thought to have originated through HGT [17], but since the sequences all group with other orchid sequences, an origin through ancient intracellular transfer seems more plausible. In *Gastrodia*, however, a few MTPTs are best explained by an origin through HGT [18]. Some of the MTPTs in *Thismia angustimitra* also show similarity to mitochondrial sequences from various eudicots suggesting that they are not results of direct plastid to mitogenome HGT events, but could be transfers between donor and recipient mitogenomes following earlier events of intracellular transfer in the donor.

Due to the degraded nature of the *Thismia* MTPTs compared to intact plastid sequences, neither the number of insertion events nor the potential donors can be precisely determined. As an example, five MTPTs stem from relatively closely located regions of the plastome (*psaB*-*psaA*-*ycf3*) and could potentially originate from a single transfer of a longer sequence, even if the individual MTPTs show highest similarity to different eudicots. Similarity of the MTPTs is low (less than 75%), and a very large number of matches to diverse taxa have almost equal similarity scores. Placing MTPTs that are potentially of foreign origin in a phylogenetic context is hampered by the short length and degraded nature of most MTPTs. However, an attempt using the longest MTPT, including part of *psaA*, provides strong support for an origin within Anacardiaceae in the Sapindales, as suggested by the similarity test. Anacardiaceae is well represented in the Thailand flora making HGT possible, but the degraded MTPT may stem from a more ancient event of HGT into an ancestral species of *Thismia*.

Despite the limitations of attempting to assign sequence homology based on just measures of similarity or approaches using types of alien indices, the large number of intergenic sequence regions showing similarity to intergenic mitochondrial sequence in other angiosperms may further suggest that horizontal transfer has occurred. Intergenic mitochondrial DNA generally has a very fast turn-over rate, e.g., in orchids less than 5% of the intergenic sequence have retained detectable similarity between genera [16]. Thus, it is not surprising that only few regions with best similarity to other members of Dioscoreaceae or monocots at large were detected in *Thismia angustimitra*. However, the large number sequence regions with high similarity to various eudicots is unexpected for a mycoheterotrophic plant albeit intergenic sequences have only rarely been investigated. In *Gastrodia*, a small fraction of the complete mitochondrial genomes confined to geneless chromosomes was analyzed and a few regions were thought to be derived from mitochondrial intergenic regions of other angiosperms [16].

While high similarity between sequences from distantly related taxa may suggest that transfer has occurred, it does not reveal the direction of transfer. Placing the sequences in a larger phylogenetic framework would be ideal, but the erratic occurrence of most intergenic sequence regions hampers a such approach. While the large number of sequence regions scattered over all chromosomes and exhibiting similarity to a wide range of taxa, may suggest insertion into *Thismia*, we propose that some of the identified regions are more likely transferred from *Thismia* to other eudicots. This could e.g. apply to regions of chromosome 5, where closely located sequences are spread over large parts of the mitogenomes of *Pityranthe trichosperma* and *Aeginetia indica*, respectively. The alternative, having distant sequences transferred to closely joint regions, seems unlikely. However, nothing is known about recombinational activity of these taxa, and a potential transfer could have involved related, even ancestral species with different sequence topology.

The long regions in chromosome 2 of very high sequence similarity to species of Orobanchaceae may provide the best evidence of horizontal transfer. In particular, two regions spanning more than 7,000 bp are 98–99% similar to *Christisonia kwangtungensis*, thus suggesting a fairly recent transfer, and substantial parts of the entire chromosome are highly similar to this or other species of Orobanchaceae. Synteny between sequence regions is limited and may as above suggest that *Thismia* is the donor, but again there is no knowledge about recombinational activity of the species of Orobanchaceae. The mitogenome of *Christisonia kwangtungensis* is described as consisting of three circular chromosomes [58], but could potentially have other configurations. Thus, the direction of a potential transfer cannot be determined with certainty.

The same applies to the small, geneless chromosome 21 where 41% of the sequence show moderate similarity to diverse species of Malvaceae. This may suggest that the entire chromosome either originates from an ancient transfer from a taxon within Malvaceae, but transfer in the opposite direction cannot be ruled out. However, the discovery of this Malvaceae-like chromosome may support the circle-mediated HGT model, which describes how foreign inserted DNA can form autonomous, circular chromosomes even when donor DNA is not circular [59, 60].

The large number of sequence regions potentially transferred between *Thismia* and Orobanchaceae is noteworthy. Several of the regions have high similarity and the best matching taxa are geographically close. *Thismia angustimitra* and *Aeginetia indica* co-occur in Thailand and Laos, and while *Christisonia kwangtungensis* is confined to southeastern China other yet unsequenced species of *Christisonia* have distribution areas overlapping with that of *Thismia angustimitra*. While frequent uptake of foreign mitochondrial DNA is well-known for parasitic plants, including member of the Orobanchaceae, most sequence transfers are assumed to involve host plant DNA [8], and to our knowledge uptake of mitochondrial DNA from mycoheterotrophic plants has not previously been found. Irrespective of the direction of transfer it is possible that the transfer can be fungus-mediated. Even if fungal DNA is not being transferred, fungal mycorrhizal partners might potentially still act as bridges between mycoheterotrophs and both autotrophic and parasitic plants. The known fungal partners of *Thismia* are species of *Rhizophagus* (Glomeraceae), some of which also form regular mycorrhizal associations with autotrophic plants [61]. Association between glomeromycetes and parasitic plants including species of Orobanchaceae is also well established [62]. Thus, a physical network that may facilitate transfer not just between mycoheterotrophic and autotrophic plants, but even between mycoheterotrophs and parasites, is likely to exist.

## Conclusions and future prospects

The loss of photosynthesis in heterotrophic plants is coupled with degradation of the plastome, and *Thismia* is no exception. In contrast, mitogenomes are usually less affected by that particular lifestyle, and accordingly, the gene content in *T. angustimitra* is unaffected. The multichromosomal composition of the mitogenome found in *T. angustimitra* is neither uncommon among angiosperms in general nor restricted to heterotrophic plants. However, the low number of repeats and very little observable recombinational activity are unusual and contrast with the suggestion that heterotrophic plants in general should have increased recombination activity compared with other plants [22]. However, complete mitogenome data from Thismiaceae is restricted to *T. angustimitra*, severely limiting evolutionary inferences and calling for more studies of mitogenome evolution is the clade.

As a general consequence of plastome reduction, intracellular sequence transfer from the plastid into the mitogenome is reduced, but the mitogenome may still contain plastid sequences either vertically inherited from an ancestor with a less reduced plastome or stemming from HGT. Accordingly, the mitogenome of *T. angustimitra* seems to include only a few sequences originating through intracellular transfer, but surprisingly other inserted sequences are best explained as derived through HGT. Even a large number of intergenic sequence regions suggests that transfer of mitochondrial sequences has taken place in both directions between *Thismia* and other angiosperms including parasitic Orobanchaceae. While HGT is observed rather frequently in haustorial parasites, it has rarely been observed in mycoheterotrophic plants, with only a few orchids providing evidence. The discovery of potential HGT also in *T. angustimitra* suggests that the phenomenon may be less uncommon in mycoheterotrophs than previously thought, but additional studies both in Thismiaceae and other lineages of mycoheterotrophic plants are strongly needed. An obvious caveat of the current study is its heavy reliance on sequence similarity to support hypotheses of HGT. Thus, it will be highly relevant to apply the recently published software, HGTScanner [63], specifically designed to detect HGT in plant mitochondrial genomes on *Thismia* data.

The potentially horizontally transferred sequences in both *Thismia* and orchids are not fungal, host DNA, but stem from other angiosperms. Whether transfer takes place in a flow from autotrophic or parasitic angiosperms through mycorrhizal fungi and into mycoheterotrophic plants or vice versa, or through some other mechanism, remains unknown but deserves future attention.

## Supplementary information

Below is the link to the electronic supplementary material.

Supplementary Material 1

Supplementary Material 2

Supplementary Material 3

Supplementary Material 4

## Data Availability

The plastome sequence of T. angustimitra is deposited in NCBI GenBank (https://www.ncbi.nlm.nih.gov/) under accession number PZ365427, and the mitogenome sequences are deposited under accession numbers PZ405831–PZ405852.
