# Supplementary material for: Characterization of the organelle genomes of mycoheterotrophic Thismia angustimitra and evidence suggesting horizontal gene transfer
Source: BMC Genomics. 2026 Nov 5;27:923. doi: 10.1186/s12864-026-13542-9 (PMC13741764; doi:10.1186/s12864-026-13542-9)
Supplement: Supplementary file 1 [file 12864_2026_13542_MOESM1_ESM.docx]

Additional file 1. Table of NCBI GenBank accession numbers for *psaA* sequences used for phylogenetic analysis.

| **Taxon** | **Accession number** |
| --- | --- |
| *Acorus gramineus* | MN175740 |
| *Aglaia odorata* | NC048994 |
| *Aletris fauriei* | NC033412 |
| *Amborella trichopoda* | NC005086 |
| *Ancistrocladus tectorius* | NC041258 |
| *Brassica carinata* | NC059807 |
| *Bupleurum scorzonerifolium* | NC041258 |
| *Bursera palmeri* | NC085820 |
| *Carludovica palmata* | NC026786 |
| *Citrus limonia* | NC065755 |
| *Convallaria pseudomajalis* | NC062624 |
| *Cotinus coggygria* | ON167901 |
| *Dioscorea rotundata* | NC024170 |
| *Helianthus salicifolius* | NC057461 |
| *Helleborus atrorubens* | NC062141 |
| *Hibiscus cannabinus* | NC045873 |
| *Japanolirion osense* | NC036154 |
| *Koelreuteria paniculata* | NC037176 |
| *Lilium fargesii* | NC033908 |
| *Magnolia mexicana* | MN700657 |
| *Mangifera quadrifida* | PQ568954 |
| *Metanarthecium luteoviride* | NC029214 |
| *Nymphaea atrans* | NC060672 |
| *Oxalis corymbosa* | NC048890 |
| *Pachysandra terminalis* | OQ397654 |
| *Pelargonium myrrhifolium* | NC_031201 |
| *Rhus hypoleuca* | MW238820 |
| *Russelia equisetiformis* | NC072594 |
| *Senna nomame* | PX097887 |
| *Solanum angustifidum* | NC062517 |
| *Tofieldia thibetica* | NC029813 |
| *Toxicodendron vernicifluum* | OQ168006 |
| *Typha orientalis* | NC050678 |
