# Supplementary material for: Characterization of the organelle genomes of mycoheterotrophic Thismia angustimitra and evidence suggesting horizontal gene transfer
Source: BMC Genomics. 2026 Nov 5;27:923. doi: 10.1186/s12864-026-13542-9 (PMC13741764; doi:10.1186/s12864-026-13542-9)
Supplement: Supplementary file 2 [file 12864_2026_13542_MOESM2_ESM.docx]

**Additional file 2.** Individual mitochondrial chromosomes of *Thismia angustimitra* shown as original assembly graphs visualized by Bandage.

**Notes:**

Chromosomes including only tRNA genes (chrom 8, chrom 13), only the short nad1 exon 4 (chrom 10) or no genes at all (chrom15, chrom 16, chrom 19, chrom 21, chrom 22) were not visualized by Bandage.

The length of chromosomes indicated here are slightly too long. Sequence ends merged by circularization are included twice. Table 2 reports the corrected lengths following high stringency read mapping.

Chromosomes 4 and 7 share Repeat 2. High stringency mapping of paired reads suggests that the two chromosomes only exist as individual entities. Partial overlap with Repeat 1 complicates assembly.
