# Supplementary material for: Characterization of the organelle genomes of mycoheterotrophic Thismia angustimitra and evidence suggesting horizontal gene transfer
Source: BMC Genomics. 2026 Nov 5;27:923. doi: 10.1186/s12864-026-13542-9 (PMC13741764; doi:10.1186/s12864-026-13542-9)
Supplement: Supplementary file 3 [file 12864_2026_13542_MOESM3_ESM.docx]

Additional file 4. Table of intergenic sequence regions of the Thismia angustimitra mitogenome with similarity to other taxa. Best matches >200 bp are recorded.

| **Position** | **Length (bp/%)** | **Similarity (%)** | **Best matching species (family), sequence location** |
| --- | --- | --- | --- |
| **Chrom 1** |  |  |  |
| 3360-3835 | 476 | 71 | *Tetradium ruticarpum* (Rutaceae), MT intergenic |
| 3847-4111 | 265 | 71 | *Cercis canadensis* (Fabaceae), MT intergenic |
| 5023-5699 | 677 | 68 | *Durio zibethinus* (Malvaceae), MT intergenic |
| 10042-10648 | 607 | 72 | *Polygonatum kingianum* (Asparagaceae), MT intergenic |
| 18432-19271 | 840 | 86 | *Zingiber officinale* (Zingiberaceae), MT intergenic |
| 19486-20265 | 780 | 90 | *Zingiber officinale* (Zingiberaceae), MT intergenic |
| 20368-20826 | 459 | 90 | *Zizyphus jujuba* (Rhamnaceae), MT intergenic |
| 20995-21360 | 366 | 80 | *Durio zibethinus* (Malvaceae), MT intergenic |
| 23534-23846 | 313 | 76 | *Antiaris toxicaria* (Moraceae), MT intergenic |
| 38239-39736 | 1498 | 84 | *Diospyros oleifera* (Ebenaceae), MT intergenic |
| 43591-44297 | 707 | 89 | *Sapindus mukorossi* (Sapindaceae), MT intergenic |
| 44307-44805 | 499 | 85 | *Sapindus mukorossi* (Sapindaceae), MT intergenic |
| 46951-47706 | 756 | 86 | *Sapindus mukorossi* (Sapindaceae), MT intergenic |
| 49191-49499 | 309 | 75 | *Polygonatum cyrtonema* (Asparagaceae), MT intergenic |
| 49762-50029 | 268 | 79 | *Delonix regia* (Fabaceae), MT intergenic |
| 52726-53593 | 868 | 74 | *Pityranthe trichosperma* (Malvaceae), MT intergenic |
| 63965-64165 | 201 | 69 | *Vatica mangachapoi* (Dipterocarpaceae), MT intergenic |
| 65296-65681 | 386 | 79 | *Vatica mangachapoi* (Dipterocarpaceae), MT intergenic |
| 68636-68870 | 235 | 77 | *Psittacanthus palmeri* (Loranthaceae), MT intergenic |
| 69176-69424 | 249 | 80 | *Rubus armeniacus* (Rosaceae), MT intergenic |
| 70276-70531 | 256 | 84 | *Calophyllum soulattri* (Calophyllaceae), MT intergenic |
| 70924-71184 | 261 | 86 | *Apocynum venetum* (Apocynaceae), MT intergenic |
| 73312-73718 | 407 | 81 | *Firmiana danxiaensis* (Malvaceae), MT non-annotated |
| 81199-81413 | 215 | 90 | *Koelreutia paniculate* (Sapindaceae), MT intergenic |
| 89263-89464 | 202 | 83 | *Microcos paniculate* (Malvaceae), MT intergenic |
| 90905-91229 | 325 | 84 | *Panax ginseng* (Araliaceae), MT intergenic |
| 92496-93196 | 701 | 80 | *Musa balbisiana* (Musaceae), MT intergenic |
| 93199-93418 | 220 | 83 | *Galeola lindleyana* (Orchidaceae), MT intergenic |
| **Total length** | **13346/14** |  |  |
|  |  |  |  |
| **Chrom 2** |  |  |  |
| 1831-2513 | 683 | 99 | *Christisonia kwangtungensis* (Orobanchaceae), MT chrom 3, intergenic |
| 9875-13489 | 3614 | 98 | *Christisonia kwangtungensis* (Orobanchaceae), MT chrom 1, intergenic |
| 13491-16884 | 3394 | 99 | *Christisonia kwangtungensis* (Orobanchaceae), MT chrom 1, intergenic |
| 17526-18203 | 678 | 92 | *Christisonia kwangtungensis* (Orobanchaceae), MT chrom 3, intergenic |
| 18193-18432 | 240 | 100 | *Christisonia kwangtungensis* (Orobanchaceae), MT chrom 3, intergenic |
| 18425-19068 | 644 | 97 | *Christisonia kwangtungensis* (Orobanchaceae), MT chrom 3, intergenic |
| 19194-19940 | 747 | 95 | *Christisonia kwangtungensis* (Orobanchaceae), MT chrom 3, intergenic |
| 28696-29204 | 509 | 80 | *Amborella trichopoda* (Amborellaceae), MT intergenic |
| 29516-29742 | 227 | 90 | *Amborella trichopoda* (Amborellaceae), MT intergenic |
| 32879-33551 | 673 | 75 | *Dolichandrone spathacea* (Bignoniaceae), MT intergenic |
| 33673-34515 | 843 | 83 | *Curcuma longa* (Zingiberaceae), MT intergenic |
| 37968-39273 | 1306 | 98 | *Christisonia kwangtungensis* (Orobanchaceae), MT chrom 2 and 3, intergenic |
| 39261-40414 | 1154 | 96 | *Dolichandrone spathacea* (Bignoniaceae), MT intergenic |
| 40567-41013 | 447 | 70 | *Christisonia kwangtungensis* (Orobanchaceae), MT chrom 2, intergenic |
| 42375-43435 | 1061 | 89 | *Aeginetia indica* (Orobanchaceae), MT intergenic |
| 43181-44131 | 951 | 91 | *Alectra orobanchoides* (Orobanchaceae), MT intergenic |
| 44021-44689 | 669 | 95 | *Aeginetia indica* (Orobanchaceae), MT intergenic |
| 45355-46704 | 1350 | 99 | *Christisonia kwangtungensis* (Orobanchaceae), MT chrom 1, intergenic |
| **Total length** | **18805/40** |  |  |
|  |  |  |  |
| **Chrom 3** |  |  |  |
| 1446-2276 | 831 | 82 | *Pityranthe trichosperma* (Malvaceae), MT intergenic |
| 2299-2542 | 244 | 80 | *Senegalia pennata* (Fabaceae), MT intergenic |
| 2951-4012 | 1062 | 85 | *Diospyros blancoi* (Ebenaceae), MT intergenic |
| 10657-11109 | 453 | 67 | *Trichopus zeylanicus* (Dioscoreaceae), MT intergenic |
| 14761-15145 | 385 | 73 | *Mangifera sylvatica* (Anacardiaceae), MT intergenic |
| 15295-16031 | 737 | 80 | *Symplocos tanakana* (Symplocaceae), MT intergenic |
| 17095-17422 | 328 | 81 | *Theobroma cacao* (Malvaceae), MT intergenic |
| 28696-29311 | 616 | 79 | *Diospyros blancoi* (Ebenaceae), MT intergenic |
| 35871-36580 | 710 | 90 | *Pityranthe trichosperma* (Malvaceae), MT intergenic |
| 36595-36864 | 270 | 86 | *Curcuma kwangsiensis* (Zingiberaceae), MT intergenic |
| 36889-37356 | 468 | 85 | *Mangifera sylvatica* (Anacardiaceae), MT intergenic |
| **Total length** | **6104/16** |  |  |
|  |  |  |  |
| **Chrom 4** |  |  |  |
| 1638-2060 | 423 | 68 | *Polygala serpyllifolia* (Polygalaceae), MT intergenic |
| 2515-2945 | 431 | 73 | *Mangifera sylvatica* (Anacardiaceae), MT intergenic |
| 9817-10229 | 413 | 67 | *Dioscorea composita* (Dioscoreaceae), MT intergenic |
| 11060-11336 | 277 | 74 | *Cocos nucifera* (Arecaceae), MT intergenic |
| 13853-14241 | 389 | 71 | *Durio zibethinus* (Malvaceae), MT intergenic |
| 17916-18148 | 233 | 85 | *Eucalyptus urophyllus* X *E. grandis* (Myrtaceae), MT intergenic |
| 18239-18575 | 337 | 82 | *Gelsemium elegans* (Gelsemiaceae), MT intergenic |
| 21159-21859 | 701 | 82 | *Diospyros blancoi* (Ebenaceae), MT intergenic |
| 23573-23972 | 400 | 80 | *Vatica mangachapoi* (Dipterocarpaceae), MT intergenic |
| 24974-25306 | 333 | 74 | *Vatica mangachapoi* (Dipterocarpaceae), MT intergenic |
| 27704-27955 | 252 | 83 | *Koelreuteria paniculata* (Sapindaceae), MT intergenic |
| 28903-29187 | 285 | 69 | *Sanguisorba officinalis* (Rosaceae), MT intergenic |
| 29385-29733 | 349 | 84 | *Pterospermum kingtungense* (Malvaceae), MT intergenic |
| 30882-31245 | 364 | 78 | *Firmiana danxiaensis* (Malvaceae), MT intergenic |
| 31693-31920 | 228 | 84 | *Diospyros blancoi* (Ebenaceae), MT intergenic |
| **Total length** | **5415/17** |  |  |
|  |  |  |  |
| **Chrom 5** |  |  |  |
| 29-239 | 211 | 88 | *Ajuga repens* (Lamiaceae), MT intergenic |
| 6008-6363 | 356 | 82 | *Pityranthe trichosperma* (Malvaceae), MT intergenic |
| 6521-7102 | 582 | 82 | *Pityranthe trichosperma* (Malvaceae), MT intergenic |
| 8477-8721 | 395 | 80 | *Pityranthe trichosperma* (Malvaceae), MT intergenic |
| 9339-10394 | 1056 | 87 | *Pityranthe trichosperma* (Malvaceae), MT intergenic |
| 10396-11656 | 1261 | 96 | *Aeginetia indica* (Orobanchaceae), MT intergenic |
| 11729-12050 | 322 | 87 | *Aeginetia indica* (Orobanchaceae), MT intergenic |
| 12183-13094 | 912 | 92 | *Aeginetia indica* (Orobanchaceae), MT intergenic |
| 13142-13493 | 352 | 97 | *Aeginetia indica* (Orobanchaceae), MT intergenic |
| 15069-15644 | 576 | 84 | *Diospyros oleifera* (Ebenaceae), MT intergenic |
| 17723-18008 | 286 | 68 | *Berberis vulgaris* (Berberidaceae), MT non-annotated |
| 18010-18230 | 221 | 95 | *Zingiber officinale* (Zingiberaceae), MT intergenic |
| **Total length** | **6530/30** |  |  |
|  |  |  |  |
| **Chrom 6** |  |  |  |
| 489-1350 | 862 | 84 | *Dioscorea composita*, (Dioscoreaceae), MT intergenic |
| 8586-9030 | 445 | 69 | *Syndiclis anlungensis* (Lauraceae), MT intergenic |
| 10466-11176 | 227 | 83 | *Stemona tuberosa* (Stemonaceae), MT intergenic |
| 11476-11857 | 382 | 72 | *Caryodaphnopsis henryi* (Lauraceae), MT intergenic |
| 15919-16209 | 291 | 71 | *Dioscorea composita*, (Dioscoreaceae), MT intergenic |
| **Total length** | **2207/13** |  |  |
|  |  |  |  |
| **Chrom 7** |  |  |  |
| 4566-4801 | 236 | 89 | *Firmiana danxiaensis* (Malvaceae), MT intergenic |
| 4864-5799 | 936 | 86 | *Pityranthe trichosperma* (Malvaceae), MT intergenic |
| 9603-9816 | 214 | 88 | *Tilia mandschurica* (Malvaceae), MT intergenic |
| 13116-13216 | 201 | 73 | *Dioscorea composita* (Dioscoreaceae), MT intergenic |
| 13430-13877 | 448 | 67 | *Dioscorea composita* (Dioscoreaceae), MT intergenic |
| **Total length** | **2025/12** |  |  |
|  |  |  |  |
| **Chrom 8** |  |  |  |
| 1458-1942 | 485 | 79 | *Zingiber officinalis* (Zingiberaceae), MT intergenic |
| 6291-7404 | 1114 | 97 | *Christisonia kwantungensis* (Orobanchaceae), MT chrom 3, intergenic |
| 7554-9031 | 1478 | 99 | *Christisonia kwantungensis* (Orobanchaceae), MT chrom 3, intergenic + *trnK* |
| 12722-13334 | 1313 | 85 | *Pediomelum tenuiflorum* (Fabaceae), MT *ccmFc* intron |
| **Total length** | **4390/27** |  |  |
|  |  |  |  |
| **Chrom 9** |  |  |  |
| 793-1005 | 213 | 81 | *Pinellia ternata* (Araceae), MT intergenic |
| 4673-6273 | 1601 | 71 | *Xerophyllum tenax* (Melanthiaceae), MT non-annotated |
| 9497-9756 | 260 | 79 | *Durioi zibethinus* (Malvaceae), MT intergenic |
| 12072-12728 | 657 | 76 | *Sapindus mukorossi* (Sapindaceae), MT intergenic |
| 12738-13652 | 915 | 80 | *Sapindus mukorossi* (Sapindaceae), MT intergenic |
| **Total length** | **3646/26** |  |  |
|  |  |  |  |
| **Chrom 10** |  |  |  |
| 1373-1601 | 229 | 89 | *Cercis chinensis* (Fabaceae), MT intergenic |
| 1959-2506 | 548 | 85 | *Tamarindus indica* (Fabaceae), MT intergenic |
| 2517-2813 | 297 | 81 | *Nekemia grossodentata* (Vitaceae), MT intergenic |
| 2963-3245 | 283 | 83 | *Castanospermum australe* (Fabaceae), MT intergenic |
| 5847-7295 | 1449 | 71 | *Elais guineensis* (Arecaceae), MT intergenic |
| 10633-10903 | 271 | 83 | *Hydrangea chinensis* (Hydrangeaceae), MT intergenic |
| 12003-12262 | 260 | 78 | *Elais guineensis* (Arecaceae), MT intergenic |
| **Total length** | **3337/24** |  |  |
|  |  |  |  |
| **Chrom 11** |  |  |  |
| 4787-5575 | 789 | 67 | *Phoenix dactylifera* (Arecaceae), MT intergenic + *trnY* |
| 11385-11918 | 534 | 72 | *Ballota nigra* (Lamiaceae), MT non-annotated |
| 13170-13549 | 380 | 84 | *Koelreutia paniculata* (Sapindaceae), MT intergenic |
| **Total length** | **1703/14** |  |  |
|  |  |  |  |
| **Chrom 12** |  |  |  |
| 179-1116 | 938 | 71 | *Primulina huaijiensis* (Gesneriaceae), N uncharacterized protein |
| 6354-6600 | 257 | 92 | *Cercis canadensis* (Fabaceae), MT intergenic |
| 7963-9114 | 1152 | 88 | *Diospyros kaki* (Ebenaceae), MT intergenic |
| **Total length** | **2347/20** |  |  |
|  |  |  |  |
| **Chrom 13** |  |  |  |
| 2363-2775 | 413 | 78 | *Ribes rubrum* (Grossulariaceae), MT non-annotated |
| 5039-5550 | 512 | 73 | *Calophyllum soulattri* (Calophyllaceae), MT intergenic + *trnE* |
| 6024-6573 | 550 | 86 | *Spondias mombin* (Anacardiaceae), MT intergenic |
| **Total length** | **1475/13** |  |  |
|  |  |  |  |
| **Chrom 14** |  |  |  |
| 1688-2108 | 421 | 76 | *Firmiana danxiaensis* (Malvaceae), MT intergenic |
| 7359-7952 | 594 | 73 | *Cocos nucifera* (Arecaceae), MT intergenic |
| 11443-247 | 345 | 91 | *Diospyros oleifera* (Ebenaceae), MT intergenic |
| **Total length** | **1360/12** |  |  |
|  |  |  |  |
| **Chrom 15** |  |  |  |
| 5852-6853 | 1002 | 85 | *Pityranthe trichosperma* (Malvaceae), MT intergenic |
| 3182-4394 | 1213 | 80 | *Mangifera longipes* (Anacardiaceae), MT intergenic |
| 4432-4828 | 397 | 85 | *Guilandina minax* (Fabaceae), MT intergenic |
| 6266-6557 | 292 | 76 | *Guilandina minax* (Fabaceae), MT intergenic |
| **Total length** | **2904/28** |  |  |
|  |  |  |  |
| **Chrom 16** |  |  |  |
| 98-591 | 494 | 70 | *Robinia pseudoacasia* (Fabaceae), MT intergenic |
| 1556-2132 | 577 | 71 | *Ligustrum vulgare* (Oleaceae), MT not annotated |
| 2979-3252 | 274 | 75 | *Peganum harmala* (Nitrariaceae), MT intergenic |
| 6923-7135 | 213 | 88 | *Diospyros blancoi* (Ebenaceae), MT intergenic |
| **Total length** | **1558/18** |  |  |
|  |  |  |  |
| **Chrom 17** |  |  |  |
| 439-670 | 232 | 79 | *Jatropha curcas* (Euphorbiaceae), MT intergenic |
| 1170-1434 | 270 | 78 | *Musa textilis* (Musaceae), MT intergenic |
| 1632-2177 | 546 | 74 | *Sapindus mukorossi* (Sapindaceae), MT intergenic |
| **Total length** | **1048/12** |  |  |
|  |  |  |  |
| **Chrom 18** |  |  |  |
| 1418-1907 | 490 | 91 | *Koelreutia paniculata* (Sapindaceae), MT intergenic |
| 2282-2983 | 702 | 84 | *Narthecium ossifragum* (Nartheciaceae), MT non-annotated (likely *nad5* intron) |
| 5705-6087 | 383 | 75 | *Vernicia fordii* (Euphorbiaceae), MT intergenic |
| 7223-7599 | 374 | 75 | *Lagerstroemia indica* (Lythraceae), MT intergenic |
| **Total length** | **1949/23** |  |  |
|  |  |  |  |
| **Chrom 19** |  |  |  |
| 693-1078 | 386 | 81 | *Lonicera japonica* (Caprifoliaceae), MT intergenic |
| 2013-2353 | 341 | 86 | *Primulina hunanensis* (Gesneriaceae), MT intergenic |
| 3051-3332 | 282 | 92 | *Cinnamomum longepaniculatum* (Lauraceae), MT intergenic |
| 3529-3773 | 245 | 90 | *Cercis canadensis* (Fabaceae), MT intergenic |
| 5574-5779 | 206 | 70 | *Primulina hunanensis* (Gesneriaceae), MT intergenic |
| **Total length** | **1460/19** |  |  |
|  |  |  |  |
| **Chrom 20** |  |  |  |
| 2497-3337 | 841 | 72 | *Mangifera longipes* (Anacardiaceae), MT intergenic |
| 4643-5063 | 421 | 72 | *Phoenix roebelenii* (Arecaceae), MT intergenic |
| 5656-5875 | 220 | 79 | *Stemona parviflora* (Stemonaceae), MT intergenic |
| 6054-6546 | 493 | 65 | *Trichopus zeylanicus* (Dioscoreaceae), MT intergenic |
| 6622-6830 | 209 | 76 | *Trichopus zeylanicus* (Dioscoreaceae), MT intergenic |
| **Total length** | **2184/32** |  |  |
|  |  |  |  |
| **Chrom 21** |  |  |  |
| 1659-2388 | 730 | 71 | *Tilia cordata* (Malvaceae), MT not annotated |
| 2700-3137 | 438 | 78 | *Durio zibethinus* (Malvaceae), MT, intergenic |
| 4031-4453 | 423 | 83 | *Tilia cordata* (Malvaceae), MT not annotated |
| 4934-5241 | 308 | 83 | *Durio zibethinus* (Malvaceae), MT, intergenic |
| 5289-6071 | 783 | 82 | *Reevesia pubescens* (Malvaceae), MT intergenic |
| **Total length** | **2682/41** |  |  |
|  |  |  |  |
| **Chrom 22** |  |  |  |
| 124-529 | 406 | 89 | *Morus alba* (Moraceae), MT intergenic |
| 1169-1497 | 329 | 78 | *Nephelium lappaceum* (Sapindaceae), MT intergenic |
| 3417-3656 | 240 | 77 | *Medinilla magnifica* (Melastomataceae), MT ORF215 (hypothetical protein) |
| **Total length** | **975/25** |  |  |
| **All genome** | **87450/21** |  |  |
